# Supplementary material for: Sarcopenia and Ghrelin System in the Clinical Outcome and Prognosis of Gastroenteropancreatic Neuroendocrine Neoplasms
Source: Cancers (Basel). 2021 Dec 27;14(1):111. doi: 10.3390/cancers14010111 (PMC8750458; doi:10.3390/cancers14010111)
Supplement: Supplementary file 1 [file cancers-14-00111-s001.zip › cancers-1469937-supplementary.pdf]

cancers

MDPI

Article

CT-assessed sarcopenia and ghrelin system in the clinical outcome and prognosis of gastroenteropancreatic neuroendocrine neoplasms

Yiraldine Herrera Martínez[1], Carlos Alzas Teomiro[2,3], Soraya León Idougourram[2,3], María José Molina Puertas[2,3], Raquel Serrano Blanch[2,4], Justo Castaño[2,5,6], María Ángeles Gálvez Moreno[2,3], Manuel D. Gahete[2,5,6], Raúl M. Luque[2,5,6], Aura D. Herrera-Martínez[2,3].

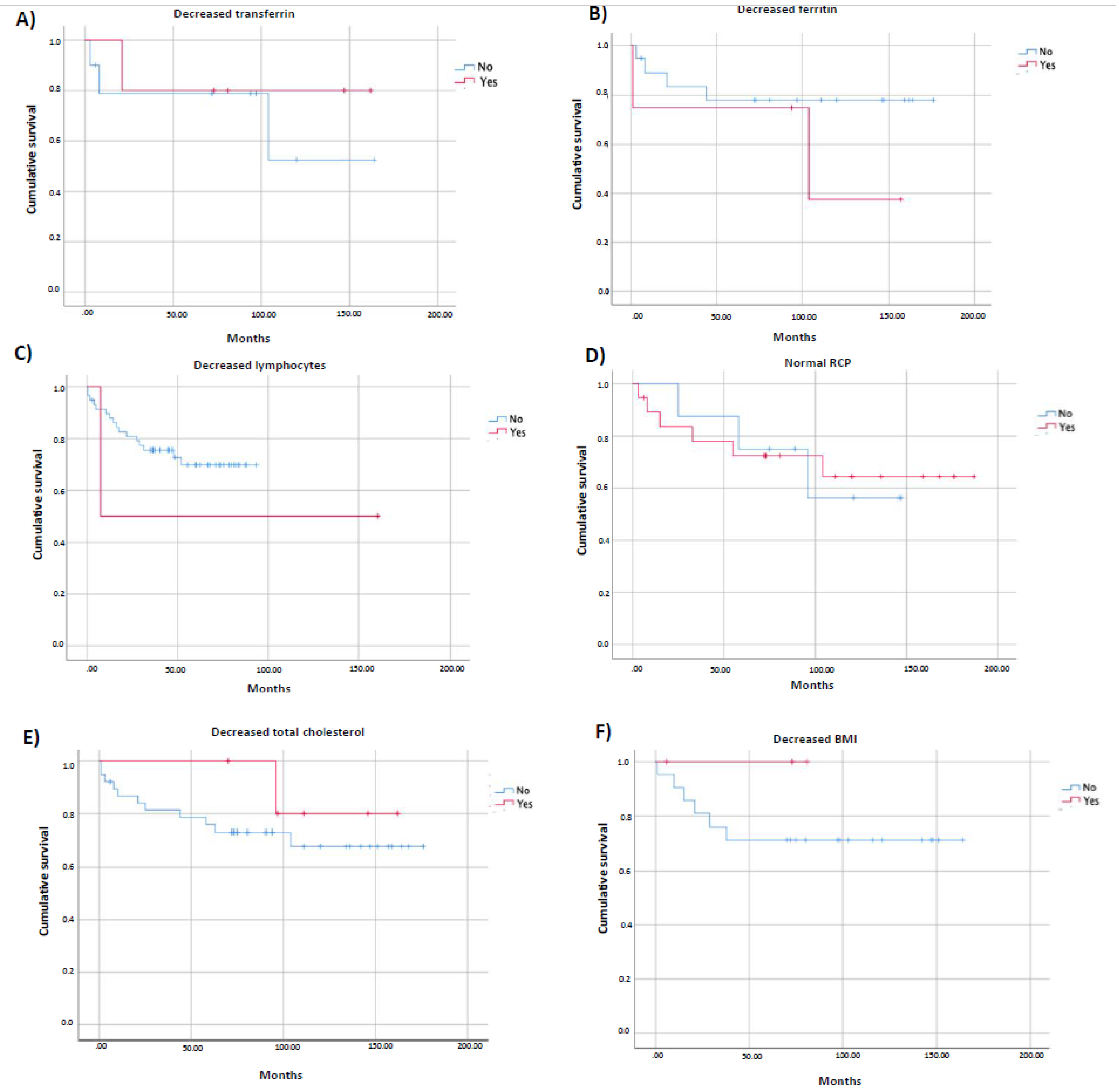

Figure S1. Cumulative survival curves for NEN patients according to nutritional parameters: (A) serum transferrin; (B) serum ferritin; (C) lymphocytes; (D) serum RCP; (E) serum total cholesterol; (F) BMI.

Table S1. Prevalence of altered nutritional parameters in patients with NEN at diagnosis according to tumor grade.

	Grade 1	Grade 2	Grade 3
**Weight loss at diagnosis**	31.8%	45.5%	22.7%
**Decreased transferrin**	44.4%	33.3%	22.2%
**Decreased ferritin**	40.6%	40%	20%
**Decreased albumin**	46.4%	39.3%	14.3%
**Decreased prealbumin**	44.4%	33.3%	22.2%
**Increased RCP**	28.6%	64.3%	7.1%
**Decreased total cholesterol**	38.7%	48.4%	12.9%
**Decreased SMI**	33.3%	63%	3.7%

Table S2. Log rank of cumulative survival curves for NEN patients according to nutritional parameters: (A) weight loss, metastasis and serum albumin levels at diagnosis; (B) BMI and other biochemical nutritional parameters.

(A)

				IQR	
		**Months**	**SD**	**5**	**95**
**Weight loss (diagnosis)**	**No**	263.6	19.6	2225.2	302
Log Rank	**Yes**	108.1	14.4	79.8	136.3
4.073 ($p = 0.04$)	**Global**	240.6	17.2	206.8	274.4
**Metastasis (diagnosis)**	**No**	245.9	18.2	210.3	281.5
Log Rank	**Yes**	136.7	14.3	108.6	164.8
3.66 ($p = 0.056$)	**Global**	214.6	15.1	185	244.2
**Decreased albumin (g/dl at diagnosis)**	**No**	142.9	11.1	121.1	164.7
Log Ra	**Yes**	65.4	20.4	25.4	105.4
3.5 ($p = 0.06$)	**Global**	135.1	11.1	113.4	156.8

(B)

Variable	Log Rank	p
BMI < 22Kg/m2	0.70	0.40
Transferrin < 215 mg/dl	0.30	0.58
Ferritin < 20 mg/dl	1.31	0.25
Lymphocytes < 900 /mm^3	0.62	0.43
Normal RCP (< 10)	0.11	0.92
Total cholesterol < 140 mg/dl	0.60	0.44
